# Comparison of Biochemical and Pathological Parameters and Parenteral Nutrition of ICU Patients Under Supervision of Dietitians and Surgeons

**DOI:** 10.3389/fnut.2021.729510

**Published:** 2021-10-07

**Authors:** Mojgan Behrad Nasab, Mohammad Esmail Akbari, Samira Rastgoo, Somayeh Gholami, Azadeh Hajipour, Nazanin Majidi, Maryam Gholamalizadeh, Samaneh Mirzaei Dahka, Saied Doaei, Mark O. Goodarzi

**Affiliations:** ^1^Department of Physical Education and Sport Sciences, Faculty of Sport Science, Central Tehran Branch, Islamic Azad University, Tehran, Iran; ^2^Cancer Research Center, Shahid Beheshti University of Medical Sciences, Tehran, Iran; ^3^Department of Clinical Nutrition and Dietetics, Research Institute Shahid Beheshti University of Medical Science, Tehran, Iran; ^4^Razi Hospital, Guilan University of Medical Sciences, Rasht, Iran; ^5^Department of Health Sciences in Nutrition, School of Health, Qazvin University of Medical Sciences, Qazvin, Iran; ^6^Department of Nutrition, Science and Research Branch, Islamic Azad University, Tehran, Iran; ^7^Student Research Committee, Cancer Research Center, Shahid Beheshti University of Medical Sciences, Tehran, Iran; ^8^Student Research Committee, Guilan University of Medical Sciences, Rasht, Iran; ^9^Reproductive Health Research Center, School of Health, Guilan University of Medical Sciences, Rasht, Iran; ^10^Division of Endocrinology, Diabetes and Metabolism, Department of Medicine, Cedars-Sinai Medical Center, Los Angeles, CA, United States

**Keywords:** parenteral nutrition, dietitian, ICU patient, ICU, PN, total parenteral nutrition, surgeon, critically ill patients

## Abstract

**Background:** Nutrient imbalance can frequently occur in patients with indications for parenteral nutrition (PN) after gastrointestinal surgery. This study aimed to compare the recommendations of a surgeon to those of a dietitian in the field of parenteral nutrition.

**Methods:** This study was performed on 256 patients undergoing gastrointestinal surgery who received PN, which included 120 patients who received PN based on recommendations of the surgeons and 136 patients who were referred to receive PN under the supervision of a dietitian in Razi Hospital in Rasht, Iran. Data on PN and clinical outcomes of the patients were collected.

**Results:** Patients under the supervision of dietitians received higher vitamin B complex and lipids and lower vitamin A and vitamin E than the surgeon-supervised patients (all *P* < 0.001). In the group receiving PN under the supervision of a surgeon, the level of blood glucose (207 vs. 182, *P* < 0.01), sodium (138 vs. 136, *P* = 0.01), potassium (3.97 vs. 3.53, *P* < 0.01), and white blood cell count (9.83 vs. 9.28, *P* < 0.01) increased significantly at the end of the PN compared to baseline. In the group receiving PN under the supervision of a dietician, the level of serum Cr (1.23 vs. 1.32, *P* = 0.04), Mg (2.07 vs. 1.84, *P* < 0.01), and pH (7.45 vs. 7.5, *P* = 0.03) significantly improved after receiving parenteral nutrition compared to baseline.

**Conclusion:** The amounts of nutrients recommended for PN by the surgeon and dietitian were different. Implementation of dietitian recommendations in critically ill patients under PN can improve patients' clinical parameters.

## Introduction

Parenteral Nutrition (PN) is applied as a method of nutrition therapy for ICU patients when bowel failure prevents adequate oral or enteral nutrition (1). After the patient is admitted to the ICU and the patient does not tolerate enteral nutrition for more than 2–3 days, it is recommended to start PN as an alternative or supplemental diet therapy (2). By preventing malnutrition and reducing stress, PN has positive effects on critical care, especially of people older than 50 years of age (3). On average, ~34,000 patients in the United States receive PN each year. (4). Parenteral nutrition can lead to a moderate increase in pre-albumin, which is one of the markers of survival in critically ill patients (5, 6). However, PN is an expensive nutritional support and may have serious side effects if not properly administered (1). As a result, it is important to provide nutritional recommendations based on standard guidelines in order to minimize nutritional complications. The American Society for Parenteral and Enteral Nutrition (ASPEN) and the European Society for Clinical Nutrition and Metabolism (ESPEN) formed special groups to encourage proper use of PN to promote benefits and reduce risks (1). PN standardization was developed by ESPEN and ASPEN to increase patient safety and clinical suitability (7).

Nutrient imbalance can frequently occur in patients after gastrointestinal surgery with indications for PN (8). Energy-protein deficiency is a common clinical problem in critically ill patients. On the other hand, the risk of overfeeding in parenteral nutrition is greater than that of enteral nutrition (2), and there is a risk of circulatory infection with increasing calorie supply in parenteral nutrition (9) The prevalence of carbohydrate metabolism disorders in critically ill patients is very high, which complicates insulin therapy and the amount of metabolic control achieved (10). High levels of blood glucose in patients with PN can lead to increased mortality (11). Parenteral nutrition can also exacerbate liver and biliary disorders (12).

Surgeons, internists, critical care medicine specialists, pharmacists, and dietitians are responsible for providing nutritional recommendations for ICU patients with PN. Dietitians may apply different nutritional recommendations compared with the other specialists based on different training and responsibilities. Applying the advice of dietitians to assess nutritional requirements and determine the amount of nutritional supplements needed can be effective in improving the health status of critically ill patients. However, some surgeons prefer to order nutritional recommendations directly and not all patients with PN indications are referred to a dietitian. No study has been done to compare the nutritional recommendations of the dietitians with surgeons and their effects on patients. So, the aim of this study was to compare the biochemical and pathological parameters and parenteral nutrition of ICU patients under supervision of surgeons or a dietician.

## Methods

### Participants

This retrospective study was performed on 256 patients with Gastroenterologic disease undergoing gastrointestinal surgery with intestinal failure and indication for TPN, which included 120 patients who received PN based on recommendations of the surgeons and 136 patients who received PN under the supervision of a single dietitian in the years 2019 and 2020 in Razi Hospital in Rasht, Iran. The sample size was estimated based on a previous similar study (13). Inclusion criteria were indication for receiving PN, age between 50 and 80 years, and consent to participate in the study. The exclusion criteria were lack of access to sufficient information on the amount of PN received and receiving enteral or oral nutrition along with PN.

### Data Collection

Age, sex, weight, height, BMI, duration of hospitalization, medical history including chronic diseases (i.e., diabetes, chronic kidney diseases, hyperlipidemia, and hypertension), diagnosed disease, pathological indices, the Acute Physiologic Assessment and Chronic Health Evaluation II (APACHE II), Glasgow coma score (GCS), and blood glucose (BG), sodium (Na), potassium (K), urea nitrogen (BUN), creatinine (Cr), white blood cell count (WBC), magnesium (Mg), albumin (Alb), calcium (Ca), and pH were extracted from the medical records before and after TPN. Information related to the nutritional recommendations in the field of TPN, including the amount and percentage of dextrose, amino acids, lipids, vitamins, and minerals were collected from ICU sheets after PN was finished. SMOFlipid® (Fresenius Kabi, United states) was used as the lipid source, which is a composite parenteral nutrition (PN) lipid, comprised of soybean oil (30%), medium-chain triglycerides (MTCs, 30%), olive oil (25%), and fish oil (15%). The mean essential fatty acid content of SMOFlipid is 35 mg/mL (range of 28 to 50 mg/mL) linoleic acid (omega-6) and 4.5 mg/mL (range of 3–7 mg/mL) α-linolenic acid (omega-3) (14). The amount of macro-nutrients administration was determined according to the patient's weight. The amount of micro-nutrients prescribed was vitamin A 50,000 IU/d, vitamin E 100 IU/d, vitamin C 500 mg/d, vitamin B complex containing vitamin B1 10 mg/d, vitamin B2 4 mg/d, vitamin B3 40 mg/d, vitamin B5 6 mg/d, and vitamin B6 4 mg/d.

### Statistical Analysis

The two groups receiving parenteral nutrition under the supervision of surgeons or a dietitian were compared in terms of demographic and pathological indicators using independent *T*-test and Chi-square methods. Also, the amounts of macronutrients and micronutrients received in the two groups were compared by independent *T*-test. The two groups were compared regarding the number of patients who received the nutrients using Chi-square and Fisher's exact test. The values of clinical and biochemical parameters before and after PN in each group were compared by paired *t*-test. All analyzes were performed using SPSS software version 21 and the significance level was considered as *P* >0.05.

### Ethical Considerations

Written consent forms were obtained from all participants or their first-degree relatives. This study was approved by the ethics committee of Shahid Beheshti University of Medical Sciences, Tehran, Iran.) code IR.SBMU.CRC.REC.1398.015).

## Results

No significant difference was found in terms of sex (males: 46.7 vs. 50.0%), age (62 ± 12 vs. 67 ± 18 years), weight (73 ± 6 vs. 75 ± 7 kg), height (165 ± 6 vs. 168 ± 6 cm), duration of hospitalization (27 ± 31 vs. 22 ± 8 days), and BMI (27 ± 2.7 vs. 26 ± 3 kg/m^2^) between dietitian-supervised and surgeon supervised groups (Table 1). In addition, no significant difference was seen between the two groups in terms of history of hypertension (50 vs. 47%), hyperlipidemia (23.5 vs. 13%), ischemic heart disease (8.8 vs. 6.7%), and hemoglobin level (9 ± 1 vs. 9 ± 1) (Table 1).

**Table 1:** Characteristics of the patients.

	**Surgeon-supervised patients (*n* = 120)**	**Dietitian -supervised patients (*n* = 136)**	***P***
Males	56 (46.7%)	68 (50.0%)	0.49
Females	64 (53.3%)	68 (50%)	
Age (y)	62 (±12)	67 (±18)	0.28
Underlying diseases			
HTN	56 (47%)	68 (50%)	0.49
HLP	16 (13%)	32 (23.5%)	0.24
IHD	8 (6.7%)	12 (8.8%)	0.56
DM	28 (23.3%)	84 (61.8%)	0.002
CKD	0 (0%)	15 (20%)	0.03
Hospitalization (day)	22 (±8)	27 (±31)	0.32
Weight (kg)	75 (±7)	73 (±6)	0.31
Height (cm)	168 (±6)	165 (±6)	0.11
APACHE II	21 (±1)	14 (±2)	<0.001
GCS	15 (± 1,5)	8 (±0.5)	<0.001
BG (mg/dl)	182 (±28)	209 (±31)	0.001
NA (mEq/L)	135 (±3)	141 (±5)	<0.001
K (mEq/L)	3 (±0.3)	4 (±0.4)	<0.001
BUN (mg/dl)	38 (±10)	47 (±14)	0.004
Cr (mg/dl)	1.11 (±0.1)	1.32 (±0.2)	<0.001
Hb (gr/dl)	9.51 (±1)	8.98 (±1)	0.06
BMI (kg/m^2^)	26 (±3)	27 (±2.7)	0.85

*HTN, hypertension; HLP, hyperlipoproteinemia; IHD, ischemic heart disease; DM, diabetes mellitus; CKD, chronic kidney disease; APACH II, The Acute Physiologic Assessment and Chronic Health Evaluation II; GCS, Glasgow coma score; BG, blood glucose; Na, sodium; K, potassium; BUN, blood urea nitrogen; Cr, creatinine; Hb, hemoglobin; BMI, body mass index*.

Dietitian-supervised patients had a higher burden of chronic diseases (79.4 vs. 53.3%, *p* = 0.025), diabetes (61.8 vs. 23.3%, *P* = 0.002), chronic kidney disease (CKD) (20 vs. 0%, *P* = 0.03), and levels of BG (209 ± 31 vs. 182 ± 28 mg/dl, *P* = 0.001), Na (141 ± 5 vs. 135 ± 3 mEq/L, *P* < 0.001), K (4 ± 0.4 vs. 3 ± 0.3 mEq/L, *P* < 0.001), BUN (47 ± 14 vs. 38 ± 10 mg/dl, *P* = 0.004), and Cr (1.32 ± 0.2 vs. 1.11 ± 0.1 mg/dl, *P* < 0.001) compared to the surgeon-supervised patients. Surgeon-supervised patients had higher APACHE II score (21 ± 1 vs. 14 ± 2, *P* < 0.001) and GCS (15 vs. 8 ± 0.5, *P* < 0.001) compared to the dietitian-supervised patients.

Regarding the percentages of the patients who received different nutrients and met the recommended amounts, the results showed that the number of patients receiving lipid (*P* < 0.001) and vitamin B complex was significantly higher in dietitian-supervised group, while the number of patients receiving vitamin A and vitamin E was significantly higher in surgeon-supervised group (Table 2). Regarding nutritional recommendations, the number of days that each patient received lipids (5.59 ± 1.13 vs. 2 ± 2.55 days, *P* < 0.001) and vitamin B complex (8.1 ± 2.8 vs. 0 days, *P* = 0.001) was higher in the dietitian-supervised group compared to the surgeon-supervised group (Table 2).

**Table 2:** Average number of days to receive nutrients and the percentages of the patients who received nutrients among patients under surgeon and dietitian recommendations.

	**Number of days to receive nutrients**			**Percentages of the patients who received nutrients**		
	**Surgeon-supervised patients (*n* = 120)**	**Dietitian -supervised patients (*n* = 136)**	***P***	**Surgeon-supervised patients (*n* = 120)**	**Dietitian -supervised patients (*n* = 136)**	***P***
Dextrose	5.27 (±0.944)	5.45 (±0.850)	0.42	116 (96.7%)	124 (91.2%)	0.36
Amino acid	5.28 (±0.960)	5.44 (±0.824)	0.47	116 (96.7%)	136 (100%)	0.47
Lipid	2 (±2.54613)	5.59 (±1.13131)	<0.001	48 (40%)	136 (100%)	<0.001
Vitamin B complex	0 (±0)	8.1 (±2.82517)	0.001	0 (0%)	120 (88.2%)	<0.001
Vitamin C	8.33 (±2.510)	8.26 (±2.863)	0.91	120 (100%)	124 (91.2%)	0.14
Vitamin A	8.17 (±2.674)	7.83 (±5.154)	0.88	116 (96.7%)	20 (14.7%)	<0.001
Vitamin E	8.20 (±2.631)	7.50 (±9.192)	0.93	120 (100%)	8 (5.9%)	<0.001

In the group receiving parenteral nutrition under the supervision of a surgeon, the level of BG (207 ± 35 vs. 182 ± 28, *P* < 0.01), sodium (138 ± 3 vs. 136 ± 3 mg/dl, P = 0.01), potassium (3.97 ± 0.4 vs. 3.53 ± 0.4, *P* < 0.01), and white blood cell count (9.83 ± 2.5 vs. 9.28 ± 2.4 10^9^/L, *P* < 0.01) increased significantly at the end of the parenteral nutrition period compared to baseline. In the group receiving parenteral nutrition under the supervision of a dietician, the level of serum Cr (1.23 ± 0.2 vs. 1.32 ± 0.2 mg/dl, *P* = 0.04), Mg (2.07 ± 0.2 vs. 1.84 ± 0.2 mg/dl, *P* < 0.01), and pH (7.45 vs. 7.5, *P* = 0.03) significantly improved after receiving parenteral nutrition compared to baseline. Serum urea, albumin and calcium levels after parenteral nutrition in the two groups were not significantly different from the baseline levels (Table 3).

**Table 3:** Comparison of Clinical outcomes of two groups at baseline and after parenteral nutrition.

	**Surgeon-supervised patients (*****n*** **=** **120)**				**Dietitian -supervised patients (*****n*** **=** **136)**			
	**Mean (±SD) at baseline**	**Mean (SD) after PN**	**Mean difference**	***P***	**Mean (SD) at baseline**	**Mean (SD) after PN**	**Mean difference**	***P***
BG (mg/dl)	182 (±28.35)	207 (±35.46)	24.29	<0.01	209 (±31.25)	208 (±43.57)	−1.94	0.81
Na (mEq/L)	136.6 (±3.1)	138.2 (±3.85)	1.6	0.01	141.79 (±5.03)	142.02 (±4.96)	0.23	0.77
K (mEq/L)	3.53 (±0.37)	3.97 (±0.41)	0.43	<0.01	3.98 (±0.45)	4.1 (±0.38)	0.11	0.23
BUN (mg/dl)	37.86 (±9.86)	36.86 (±9.53)	1	0.36	46.94 (±13.96)	44.85 (±13.75)	−2.08	0.31
Cr (mg/dl)	1.11 (±0.11)	1.31 (±0.17)	0.01	0.48	1.32 (±0.24)	1.23 (±0.23)	−0.09	0.04
WBC (109/L)	9.28 (±2.37)	9.83 (±2.55)	0.55	<0.01	8.71 (±2.46)	9.1 (±2.97)	0.38	0.14
Mg (mg/dl)	1.95 (±0.25)	1.98 (±0.21)	0.03	0.31	1.84 (±0.15)	2.07 (±0.22)	0.22	<0.01
Alb (g/dl)	2.97 (±0.31)	2.88 (±0.17)	−0.04	0.49	2.93 (±0.31)	2.89 (±0.28)	−0.04	0.49
Ca (mg/dL)	8.45 (±0.45)	8.38 (±0.64)	−0.7	0.42	7.75 (±0.45)	7.85 (±0.64)	0.1	0.45
pH	7.44 (±0.86)	7.45 (±0.73)	0.01	0.08	7.5 (±0.86)	7.45 (±0.73)	−0.05	0.03

## Discussion

In the present study, for the first time, the performance of dietitians was compared to surgeons in parenteral feeding of patients after gastrointestinal surgery. The results indicated that patients with worsening conditions were referred to a dietitian. Moreover, patients under the supervision of dietitians received higher vitamin B complex and lipids than the group under the supervision of surgeons. In the surgeon-supervised group, the patients received higher amounts of vitamin A and vitamin E than the dietitian-supervised patients. In the group receiving parenteral nutrition under the supervision of a surgeon, the level of BG, sodium, potassium, and white blood cells count increased significantly at the end of the PN compared to baseline. In the group receiving PN under the supervision of a dietician, the level of serum Cr, Mg, and pH significantly improved after receiving parenteral nutrition compared to baseline.

Providing parenteral nutrition can be vital for patients with intestinal failure, but achieving the desired amount and balance is a complicated issue and many factors such as age, degree of inflammation, number of failing organs, comorbidities, estimated length of stay, gastrointestinal function, fluids and electrolytes, and BG control must be considered in parenteral nutrition planning. Patients admitted to the ICU should receive PN within 24–48 h if they are unable to tolerate enteral nutrition.

Tignanelli et al. reported that mortality was lower in patients with nutritional counseling and that malnutrition should be prevented in order to prevent adverse consequences. Malnutrition increases the risk of disease, adverse surgical outcomes, length of stay in the hospital, and cost burden. Disease-induced stress in ICU patients may accelerate the development of malnutrition.

Patients receiving nutritional care from dietitians were reported to reach the target dietary intake faster and their clinical outcomes were improved (15). Vankrunkelsven et al. examined parenteral administration of micronutrients including phosphate, magnesium, iron and B-complex vitamins including vitamins B12, B1, and folic acid and concluded that nutrient deficiency may be related to the degree of inflammation (16).

In the study by Heyland et al., most ICU patients did not receive adequate nutritional support, especially early in their illness, and their energy and protein requirements were not correctly estimated (17).

We found that patients receiving parenteral nutrition under the supervision of a dietitian received similar dextrose compared with the patients under the supervision of a surgeon. Several previous reports indicated that receiving dextrose parenterally during the first week in the ICU leads to fewer secondary infections, less weakness, rapid recovery, and reduced patient mortality (18–21). However, hyperglycemia is an independent risk factor for short-term infection in patients undergoing surgery (14, 15). The risk of hyperglycemia as a part of the endocrine metabolic response to stress is present in almost all patients in the ICU. If the requirement for intravenous dextrose in patients is not specifically assessed and determined, it may increase the risk of hyperglycemia. In the present study, the blood sugar level of patients under the supervision of a surgeon increased significantly after receiving intravenous nutrition.

In the present study, patients receiving parenteral nutrition under the supervision of a dietitian received more lipid than patients under the supervision of a surgeon. Lipids should be considered as an integral part of PN to provide energy and ensure a supply of essential fatty acids. Providing essential fatty acids in PN using standard lipid emulsions can lead to additional clinical benefits such as reductions in both infection rate and length of hospital stay (22, 23). Various mixtures of lipid emulsions, including soybean oil, medium chain triglycerides, olive oil, and omega-3 rich fish oil, are widely available for parenteral nutrition. Omega-3 fatty acids can have beneficial immune-regulating and anti-inflammatory effects in a wide range of patients undergoing surgery (23). The addition of EPA and DHA to lipid emulsions may improve cell membrane function and inflammation, and reducing the length of stay of critically ill patients in the ICU (23). Pradelli et al. in a systematic review reported that omega-3 fatty acid-containing PN was associated with clinically significant improvement in patient outcomes (24). In the present study, SMOFlipid was used as the lipid source of TPN, which is rich in omega-3 fatty acids and higher lipid intake in the group under the supervision of a dietitian was associated with improved serum creatinine and pH levels and no increase in BG levels. However, high intake of unsaturated fatty acids in critically ill patients may be associated with side effects such as disturbed liver function or altered balances of antioxidants (25) and should be recommended according to the patient's requirements.

The results of this study indicated that dietitians may be better able to assess the nutritional requirements of critically ill patients and significantly help to improve the biochemical and pathological parameters of these patients. In line with the present study, evidence suggests that dietitians are key members of the ICU care team who can help improve patient outcomes (26). Severely ill patients who had sufficient nutritional intake were less likely to develop pneumonia, pulmonary insufficiency, gastrointestinal bleeding, or the need for mechanical ventilation (27).

The intake of vitamins A and E in the group under the supervision of the surgeon was higher and the intake of B vitamins was lower than the group under the supervision of the dietitian. Because of the risk of toxicity, fat soluble vitamins such as vitamin E and vitamin A should not be prescribed at high doses without proven deficiency. It was reported that patients with renal failure may be at risk for symptomatic vitamin A toxicity if given PN with standard retinol supplementation. However, vitamin E may reduce the length of mechanical ventilation in ICU patients (28, 29). On the other hand, critical illness in adults is characterized by absolute or relative thiamine depletion, which is associated with an almost 50% increase in mortality. Vitamin B1 is likely to be used in high-risk patients to prevent Wernicke's encephalopathy and heart failure. Moreover, administration of vitamin B1 may be used as adjunctive therapy in septic shock (30–33).

However, this study was limited to the ICU patients undergoing surgery, which makes it difficult to generalize the results to other patients. Moreover, the dietitian-supervised patients were significantly different compared with surgeon-supervised patients in terms of history of chronic diseases and pathological and biochemical parameters which may influence nutritional recommendations as well as the biochemical changes observed after TPN. In addition, individual dietary requirements of the patients were not assessed. Future longitudinal studies are needed to confirm these results and to investigate the effects of dietitian and surgeon PN recommendations on health outcomes of the patients.

## Conclusion

The results indicated that patients with worsening conditions were referred to a dietitian. Moreover, patients under the supervision of dietitians received higher vitamin B complex and lipids than the surgeon-supervised patients. In the surgeon-supervised group, the patients received higher amounts of vitamin A and vitamin E than the dietitian-supervised patient. Biochemical changes suggestive of better outcomes were observed in the dietician-supervised group. Future longitudinal studies are needed to investigate the effects of dietitian and surgeon PN recommendations on health outcomes of the patients.

## Data Availability Statement

The raw data supporting the conclusions of this article will be made available by the authors, without undue reservation. Data may be made available upon request.

## Ethics Statement

The written consent forms were obtained from all participants or their first-degree relatives. This study was approved by the Ethics Committee of Shahid Beheshti University of Medical Sciences, Tehran, Iran (code IR.SBMU.CRC.REC.1398.015). The patients/participants provided their written informed consent to participate in this study.

## Author Contributions

SD, MB, MA, MG, SR, SG, AH, and NM designed the study, involved in the data collection, analysis, and drafting of the manuscript. SMD, MOG, and SD were involved in the design of the study, analysis of the data, and critically reviewed the manuscript. All authors read and approved the final manuscript.

## Funding

Funding for this study was provided by Shahid Beheshti University of Medical Sciences, Tehran Iran (code 16415) and Guilan University of Medical Sciences, Rasht, Iran (code 578).

## Conflict of Interest

The authors declare that the research was conducted in the absence of any commercial or financial relationships that could be construed as a potential conflict of interest.

## Publisher's Note

All claims expressed in this article are solely those of the authors and do not necessarily represent those of their affiliated organizations, or those of the publisher, the editors and the reviewers. Any product that may be evaluated in this article, or claim that may be made by its manufacturer, is not guaranteed or endorsed by the publisher.
